# Supplementary material for: Rapid and Accurate Approach for Honeybee Pollen Analysis Using ED-XRF and FTIR Spectroscopy
Source: Molecules. 2021 Oct 4;26(19):6024. doi: 10.3390/molecules26196024 (PMC8512728; doi:10.3390/molecules26196024)
Supplement: Supplementary file 1 [file molecules-26-06024-s001.zip › Supp Mat. Figure S3.pdf]

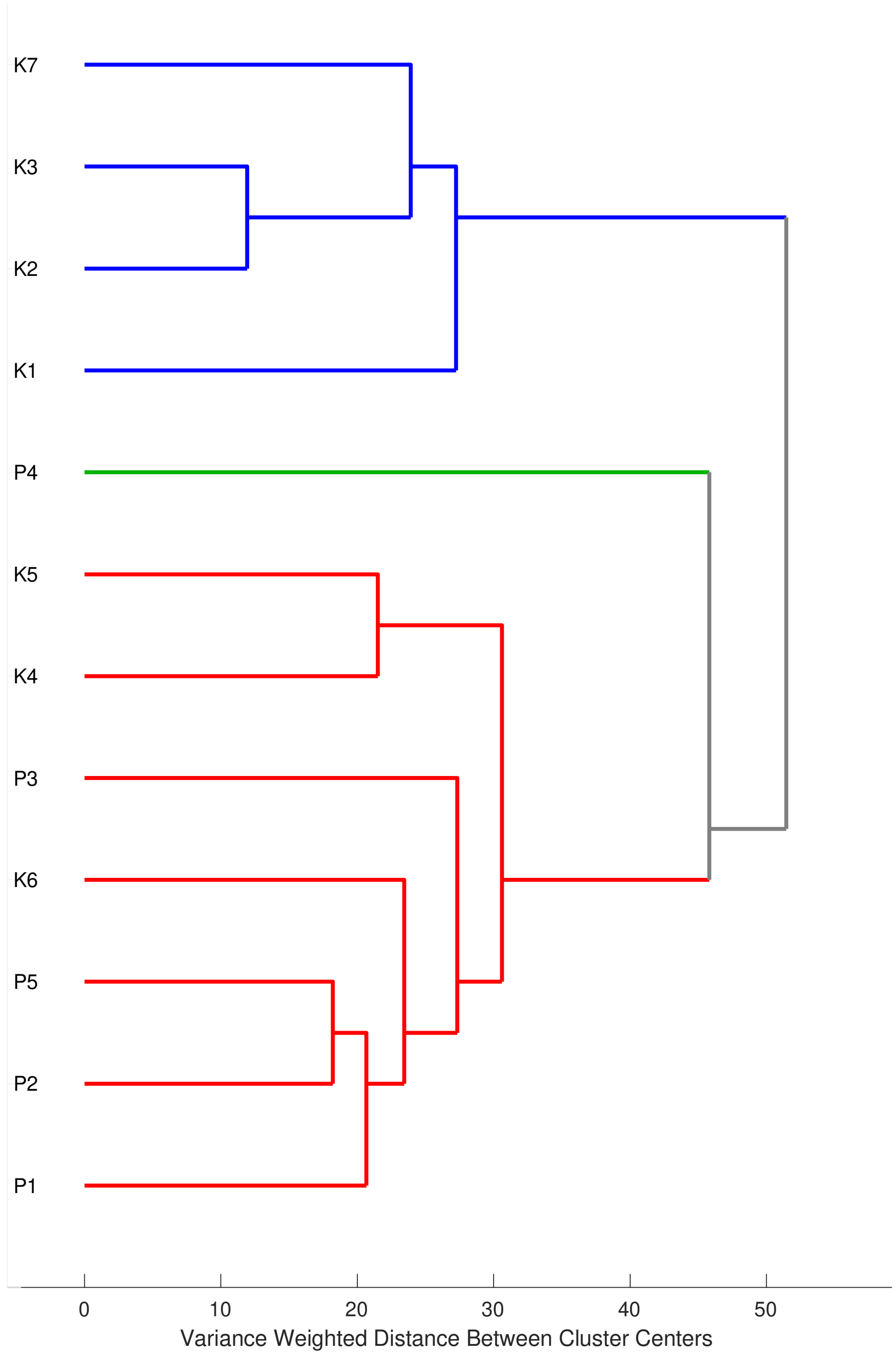

Figure S3: Dendrogram obtained by cluster analysis performed on the data from ATR–FTIR analysis for bee pollen samples K1–K7 and P1–P5.
