# Supplementary material for: Rapid and Accurate Approach for Honeybee Pollen Analysis Using ED-XRF and FTIR Spectroscopy
Source: Molecules. 2021 Oct 4;26(19):6024. doi: 10.3390/molecules26196024 (PMC8512728; doi:10.3390/molecules26196024)
Supplement: Supplementary file 1 [file molecules-26-06024-s001.zip › Supp. Mat. Figure S1.pdf]

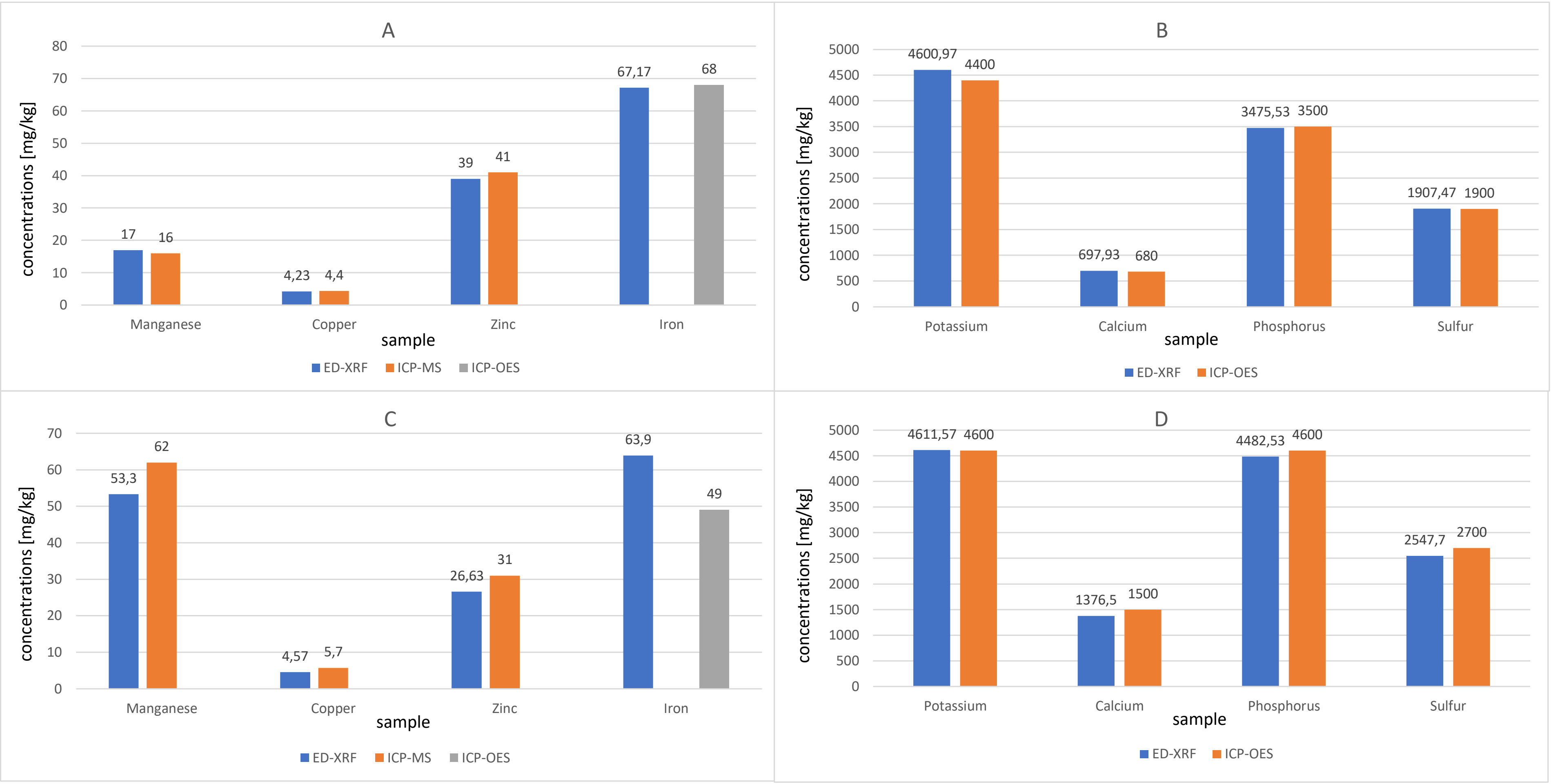

Figure S1: Comparison between results obtained by ICP-MS or ICP-OES and ED-XRF spectros-copy for sample P5 (Figure S1A,B) and P2 (Figure S1C,D).
