# Supplementary material for: Rapid and Accurate Approach for Honeybee Pollen Analysis Using ED-XRF and FTIR Spectroscopy
Source: Molecules. 2021 Oct 4;26(19):6024. doi: 10.3390/molecules26196024 (PMC8512728; doi:10.3390/molecules26196024)
Supplement: Supplementary file 1 [file molecules-26-06024-s001.zip › Supplementary Materials Table S6.pdf]

Table S6.

The comparison of the mineral concentrations in the bee pollen samples collected in different date and commercially available samples. The results were obtained using ED-XRF Fundamental Parameter method with standard sample.

Sample / Mineral	K1 (mean + SD) [mg/kg]	K2 (mean + SD) [mg/kg]	K3 (mean + SD) [mg/kg]	K4 (mean + SD) [mg/kg]	K5 (mean + SD) [mg/kg]	K6 (mean + SD) [mg/kg]	K7 (mean + SD) [mg/kg]	P1 (mean + SD) [mg/kg]	P2 (mean + SD) [mg/kg]	P3 (mean + SD) [mg/kg]	P4 (mean + SD) [mg/kg]	P5 (mean + SD) [mg/kg]
**Manganese**	25.6 ± 0.2	37.6 ± 12.3	22.6 ± 1.8	31.2 ± 1.0	18.9 ± 0.6	17.6 ± 0.6	40.1 ± 9.4	13.9 ± 1.4	53.3 ± 11.8	23.4 ± 2.2	20.5 ± 2.4	17.0 ± 0.7
**Copper**	6.6 ± 0.8	5.6 ± 0.2	5.0 ± 0.1	5.4 ± 0.5	5.6 ± 0.4	5.3 ± 0.2	5.7 ± 0.6	4.3 ± 0.2	4.6 ± 0.2	4.9 ± 0.2	4.7 ± 0.2	4.2 ± 0.4
**Zinc**	43.4 ± 4.2	35.9 ± 1.4	31.1 ± 2.6	32.7 ± 0.8	30.0 ± 0.8	33.3 ± 0.9	35.2 ± 2.7	20.9 ± 2.8	26.6 ± 0.2	32.6 ± 2.4	37.9 ± 1.8	39.0 ± 1.9
**Potassium**	5254.4 ± 645.6	4804.6 ± 254.2	5847.1 ± 571.5	7084.8 ± 413.9	4859.4 ± 316.2	4003.1 ± 229.6	5409.6 ± 137.0	3125.7 ± 165.9	4611.6 ± 379.6	4396.0 ± 167.9	4087.7 ± 197.0	4600.7 ± 71.6
**Calcium**	1402.9 ± 95.6	1144.1 ± 119.3	1858.0 ± 144.7	1339.3 ± 93.1	1345.4 ± 105.0	1359.8 ± 134.6	1444.6 ± 54.9	1569.1 ± 111.1	1376.5 ± 143.8	1068.6 ± 82.8	687.9 ± 76.6	697.9 ± 24.1
**Iron**	93.1 ± 7.9	67.1 ± 7.6	67.7 ± 1.9	125.8 ± 7.3	83.4 ± 2.9	81.9 ± 14.7	87.4 ± 6.4	160.3 ± 6.4	63.9 ± 2.2	69.0 ± 2.8	70.1 ± 2.2	67.2 ± 8.2
**Phosphorus**	5009.4 ± 542.2	4248.7 ± 394.8	6131.9 ± 549.5	5415.5 ± 196.2	3965.3 ± 244.2	3709.2 ± 160.3	5360.1 ± 100.5	2281.2 ± 140.0	4482.5 ± 490.5	4320.9 ± 207.2	3928.8 ± 191.8	3475.5 ± 124.4
**Sulfur**	2589.5 ± 206.4	2234.7 ± 204.9	3208.2 ± 252.0	2563.7 ± 124.3	2058.0 ± 25.4	2275.6 ± 75.0	2882.3 ± 161.6	1409.5 ± 174.2	2547.7 ± 385.7	2225.5 ± 94.4	2095.2 ± 88.0	1907.5 ± 44.3
