# Supplementary material for: Rapid and Accurate Approach for Honeybee Pollen Analysis Using ED-XRF and FTIR Spectroscopy
Source: Molecules. 2021 Oct 4;26(19):6024. doi: 10.3390/molecules26196024 (PMC8512728; doi:10.3390/molecules26196024)
Supplement: Supplementary file 1 [file molecules-26-06024-s001.zip › Supplementary Materials Table S7.pdf]

Table S7. Detailed information about PCA analysis performed on the data from ED-XRF and ATR-FTIR (% Variance of the particular PC, % Variance cumulative, root mean squared error of calibration – RMSEC and root mean squared error of cross validation – RMSECV).

	ED-XRF				ATR-FTIR			
	% Variance this PC	% Variance Cumulative	RMSEC	RMSECV	% Variance this PC	% Variance Cumulative	RMSEC	RMSECV
PC1	51.04	51.04	0.67	1.19	35.32	35.32	0.77	1.18
PC2	22.69	73.73	0.49	1.34	23.85	59.17	0.61	1.16
PC3	12.48	86.21	0.36	1.37	10.54	69.71	0.53	1.17
PC4	6.13	92.34	0.26	1.81	8.40	78.11	0.45	1.19
